# A Minor (<50%) Signet-Ring Cell Component Associated with Poor Prognosis in Colorectal Cancer Patients: A 26-Year Retrospective Study in China

**DOI:** 10.1371/journal.pone.0121944

**Published:** 2015-03-19

**Authors:** Yinuo Tan, Jianfei Fu, Xiaofen Li, Jiao Yang, Mengjie Jiang, Kefeng Ding, Jinghong Xu, Jun Li, Ying Yuan

**Affiliations:** 1 Dept. of Medical Oncology, 2nd Hospital of Zhejiang University School of Medicine, Hangzhou, P.R. China; 2 Dept. of Oncology, Jinhua Central Hospital, Jinhua, P.R. China; 3 Dept. of Surgical Oncology, 2nd Hospital of Zhejiang University School of Medicine, Hangzhou, P.R. China; 4 Cancer Institute, Key Laboratory of Cancer Prevention and Intervention, China National Ministry of Education, Key Laboratory of Molecular Biology in Medical Sciences, Hangzhou, Zhejiang Province, China, and The Second Affiliated Hospital, Zhejiang University School of Medicine, Hangzhou, P.R. China; 5 Dept. of Pathology, 2nd Hospital of Zhejiang University School of Medicine, Hangzhou, P.R. China; National Cancer Center, JAPAN

## Abstract

**Background:**

We performed a retrospective study to determine the cancer-specific survival of colorectal cancer patients with a component of signet-ring cells or mucin comprising < 50% of the tumor mass.

**Methods:**

A total of 2454 patients seen in our hospital from 1985 to 2011 were retrospectively studied. The patients were divided into five groups according to type of cancer: signet-ring cell carcinoma (with > 50% signet-ring cell, n = 36), partial signet-ring cell carcinoma (with < 50% signet-ring cell, n = 28), mucinous adenocarcinoma (with > 50% mucin lacking signet-ring cell, n = 267), partial mucinous adenocarcinoma (with < 50% mucin lacking signet-ring cell, n = 145), and classic adenocarcinoma (with absence of either mucin or signet-ring cell, n = 1978).

**Results:**

Patients with > 50% or < 50% signet-ring cell had the lowest 5-year survival rates (35.5% and 29.7%, respectively), followed by patients with > 50% mucin (48.8%). Patients who had partial mucinous adenocarcinoma with < 50% mucin and classic adenocarcinoma patients had the highest 5-year survival rates (64.8% and 65.3%, respectively). Stratified and multivariate analysis showed that signet-ring cell carcinoma, partial signet-ring cell carcinoma and mucinous adenocarcinoma were independent predictors of decreased survival (hazard ratio 1.699, *P* = 0.016; hazard ratio 2.182, *P* = 0.005; hazard ratio 1.532, *P* < 0.001; respectively), and partial mucinous adenocarcinoma was not (hazard ratio 1.137, *P* = 0.431).

**Conclusions:**

Patients with a component of signet-ring cells, regardless of the extent, had poor prognoses. Patients with mucinous adenocarcinoma containing >50% mucin had poor prognoses as well, whereas those with < 50% mucin had survival rates similar to those of classic adenocarcinoma patients. Therefore, in clinical practice, patients with a component of signet-ring cells, regardless of the extent, should be given significant clinical attention.

## Introduction

Adenocarcinoma is the most common pathological type of colorectal cancer (CRC), representing more than 95% of CRC cases. Its most common subsets are tubular adenocarcinoma and papillary adenocarcinoma. Mucinous adenocarcinoma (MAC) and signet-ring cell carcinoma (SRCC) are rare, with MAC accounting for 10–15% and SRCC accounting for 0.1–2.4% of CRC cases [1].

MAC and SRCC can both produce excess mucin. A unique pathologic feature of SRCC is the presence of signet-ring cells, which are single tumor cells with intracytoplasmic mucin that displace the nuclei. In comparison, MAC is characterized by abundant extracellular mucin pools produced by tumor cells.

The features of MAC and SRCC have been widely known for some time. They include the following: affecting younger patients [2–11], having more lymph node metastases [3,4,10–14] and more peritoneal metastases [1,3,5,6,8,10,12–15], and presenting at an advanced stage [3–8,10–13,15–19]. Although the poor prognosis of SRCC has been widely recognized [4,7,8,12,18,20], the prognosis of MAC remains controversial. Some researchers have demonstrated poorer survival rates among MAC patients [16,21,22], but others have not been able to replicate these results [1,23,24]. Moreover, in some cases, after stratified and multivariate analysis, MAC was found to not be an independent negative indicator for prognosis in CRC patients [2,18], leading to the hypothesis that the adverse prognostic effect of MAC could be explained by the more advanced stage at presentation.

The World Health Organization (WHO) defines SRCC (or MAC) as an adenocarcinoma in which a substantial amount (≥50% of the tumor) of signet-ring cell (or mucin) is retained within the tumor [25]. However, we have also seen colorectal adenocarcinomas with the presence of signet-ring cells (or mucin) in < 50% of the tumor. The behavior of these tumors and the overall outcomes of these patients have not been well studied. Therefore, this study was designed to characterize the prognoses of CRC patients with signet-ring cell or mucin, regardless of whether it comprised < 50% of the tumor mass. The findings may further our understanding of the role that the signet-ring cell or mucin plays in CRC.

## Materials and Methods

### Patients and data collection

This research was approved by the Ethical Committee of the Second Affiliated Hospital of Zhejiang University School of Medicine, and patient information was anonymized and de-identified prior to analysis.

In total, data for 2454 consecutive primary CRC patients were collected from the Second Affiliated Hospital of Zhejiang University School of Medicine from December 1985 to December 2011. Data including gender, age at diagnosis, date of diagnosis, tumor site, pathological diagnosis, tumor stage at the time of diagnosis were obtained by reviewing the medical records. All tumors were staged according to the TNM staging system of the American Joint Committee on Cancer (7th version, 2009). The tumor site was classified as right-sided colon (ileocecal junction, cecum, ascending colon, hepatic flexure and transverse colon), left-sided colon (splenic flexure, descending colon and sigmoid colon) or rectum. In this study, patients ≤ 35 yrs at diagnosis were referred to as young patients, the decision of 35 yrs as a cut off was based on two previously published results from our institution [26,27]. Of these 2454 patients, 2130 without distant metastasis received radical resection. Of the other 322 patients, who were diagnosed as being at stage IV at presentation or during the surgery, 32 underwent complete removal of all tumors (R0 resection), 232 had microscopic tumor cells left in the surgical margins (R1 resection), and the remaining 58 patients had either a bypass or an ileostomy (R2 resection).

The follow-up were mainly made with telephone calls. The date of last follow-up was July 2013. The reason of death was collected and recurrence rate was calculated. The main survival indexes were the cancer-specific survival (CSS), the time from operation to death caused by CRC.

### Pathologic stratification

**Fig 1:**
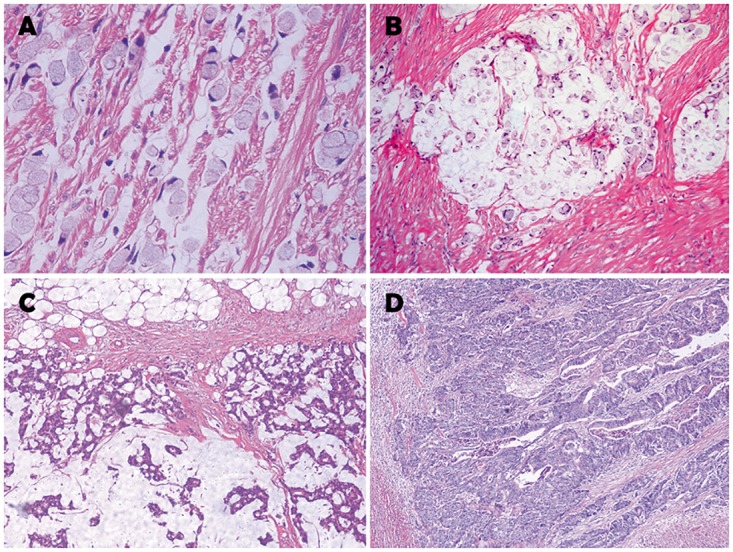
Representative histopathological images of different kinds of adenocarcinomas. (A) Signet-ring cell carcinoma. (B) Signet-ring cells floating in the pools of mucus are demonstrated. Mucinous carcinomas or classic adenocarcinomas with a minor signet-ring cell component were classified as partial signet-ring cell carcinoma. (C) Mucinous adenocarcinomas containing no signet-ring cells were classified as MAC or PMAC based on the proportion of mucin in the tumor. (D) Classic adenocarcinoma without any mucin or signet-ring cells.

Hematoxylin and eosin-stained slides of the tumors were reviewed separately by two pathologists to evaluate the percentages of signet-ring cell and mucin. According to their histological types, the patients were stratified into five groups as follows: (1) signet-ring cell carcinoma (SRCC) was defined as a lesion consisting of ≥ 50% signet-ring cells, as shown in Fig. 1A. (2) partial signet-ring cell carcinoma (PSRCC) was defined as a lesion consisting of < 50% signet-ring cells, as shown in Fig. 1B. (3) mucinous adenocarcinoma (MAC) was defined as a lesion consisting of ≥ 50% mucin and containing no signet-ring cells, as shown in Fig. 1C. (4) partial mucinous adenocarcinoma (PMAC) was defined as a lesion consisting of < 50% mucin and containing no signet-ring cells, and (5) classic adenocarcinoma (AC) was defined as the absence of any mucin or signet-ring cells, as shown in Fig. 1D.

### Statistical Analysis

The data for all categorical demographic variables were summarized with frequencies and percentages. The observed differences between histologic groups were analyzed statistically by the chi-square test. The Kaplan-Meier method was used to calculate the 5-year CSS for all groups. Finally, the log-rank test was performed to statistically evaluate the differences in survival distributions. Multivariate analyses were also performed using the Cox model. The data were processed using SPSS statistics 19.0 software.

## Results

### Clinicopathological characteristics

A total of 2454 patients with colorectal adenocarcinoma (age 18–97 yrs, median age 58.7 yrs) were evaluated, including 36 (1.5%) patients with SRCC, 28 (1.1%) patients with PSRCC, 267 (10.9%) patients with MAC, 145 (5.9%) patients with PMAC and 1978 (80.6%) patients with AC. The clinicopathological characteristics of the five groups are shown in Table 1.

**Table 1:** Clinicopathological characteristics of the five groups.

Variance	SRCC (n = 36)	PSRCC (n = 28)	MAC (n = 267)	PMAC (n = 145)	AC (n = 1978)	Total (n = 2454)
**Age**						
Interquartile range	(34,40,59)	(41,61,72)	(44,56,67)	(49,61,70)	(52,60,69)	<0.001
< = 35years old	13(36.1%)	4(14.3%)	38(14.2%)	3(2.1%)	85(4.3%)	
>35years old	23(63.9%)	24(85.7%)	229(85.8%)	142(97.9%)	1893(95.7%)	
*P* value*	<0.001	0.033	<0.001	0.277		<0.001
**Gender**						
Male	23(63.9%)	12(42.9%)	147(55.1%)	89(61.4%)	1161(58.7%)	
Female	13(36.1%)	16(57.1%)	120(44.9%)	56(38.6%)	817(41.3%)	
*P* value*	0.53	0.091	0.258	0.526		0.282
**Tumor site**						
Right-sided colon	9(25.0%)	12(42.9%)	90(33.7%)	62(42.8%)	457(23.1%)	
Left-sided colon	9(25.0%)	10(35.7%)	80(30.0%)	37(25.5%)	512(25.9%)	
Rectum	18(50.0%)	6(21.4%)	97(36.3%)	46(31.7%)	1009(51.0%)	
*P* value*	0.964	0.005	<0.001	<0.001		<0.001
**T stage**						
T1–2	4(11.1%)	0	28(10.5%)	20(13.8%)	460(23.3%)	
T3–4	14(88.9%)	28(100.0%)	239(89.5%)	124(85.5%)	1513(76.5%)	
Tx	0	0	0	1(0.7%)	6(0.3%)	
*P* value*	0.913	0.004	<0.001	0.009		<0.001
**N stage**						
N0	6(16.7%)	0	111(41.6%)	71(49.0%)	1116(56.4%)	
N1–2	29(80.6%)	28(100.0%)	139(52.1%)	72(49.7%)	769(38.9%)	
Nx	1(2.8%)	0	17(6.4%)	2(1.4%)	93(4.7%)	
*P* value*	<0.001	<0.001	<0.001	0.025		<0.001
**M stage**						
M0	25(69.4%)	22(78.6%)	218(81.6%)	129(89.0%)	1738(87.9%)	
M1	11(30.6%)	6(21.4%)	49(18.4%)	16(11.0%)	240(12.1%)	
*P* value*	0.001	0.136	0.004	0.695		0.001
**TNM stage**						
Stage I-II	6(16.7%)	0	109(40.8%)	70(48.3%)	1095(55.4%)	
Stage III-IV	30(83.3%)	28(100.0%)	158(59.2%)	75(51.7%)	883(44.6%)	
*P* value*	<0.001	<0.001	<0.001	0.098		<0.001
**Surgery**						
R0 resection	25(69.4%)	22(78.6%)	226(84.6%)	130(89.7%)	1761(89.0%)	
R1 resection	10(27.8%)	6(21.4%)	32(12.0%)	15(10.3%)	169(8.5%)	
R2 resection	1(2.8%)	0	9(3.4%)	0	48(2.4%)	
*P* value*	<0.001	0.043	0.108	0.133		<0.001
**Recurrence rate** ^**#**^	13(52.0%)	8(36.4%)	80(36.7%)	27(20.9)	396(22.8%)	<0.001

* results compared with AC. Tx and Nx patients were excluded when performing the chi-square test.

^#^Stage IV patients were excluded when calculating the recurrence rate, SRCC, signet-ring cell carcinoma; PSRCC, partial signet-ring cell carcinoma; MAC, mucinous adenocarcinoma; PMAC, partial mucinous adenocarcinoma; AC, classic adenocarcinoma.

At diagnosis, 13 of 36 (36.1%) SRCC patients were younger than 35 yrs, whereas only 4.3% (85/1978) of AC patients were considered young patients as defined in this study (*P* < 0.001). When compared with AC, significant differences were found in PSRCC (*P* = 0.033) and in MAC (*P* < 0.001), but the difference between AC and PMAC was not significant (*P* = 0.277).

There was also a significant difference between the five groups in the tumor site of the primary cancer (*P* < 0.001). Compared with AC, more right-sided tumors and fewer rectal tumors were documented in PSRCC, MAC and PMAC (*P* = 0.005, <0.001 and <0.001, respectively). The tumor site distribution of SRCC was similar to that of AC.

Significant differences were also found in the T, N, M and TNM stages with regard to tumor stage at presentation (*P* < 0.001, *P* < 0.001, *P* = 0.001, *P* < 0.001, respectively). When compared with AC patients, PSRCC, MAC and PMAC patients exhibited a higher T stage, whereas SRCC, PSRCC, MAC and PMAC patients exhibited higher N and TNM stages, with a greater likelihood of metastasis in SRCC and MAC patients. More R1 resections were performed on SRCC and PSRCC patients, compared with AC patients (*P* < 0.001).

### Overall survival and recurrence rate

**Fig 2:**
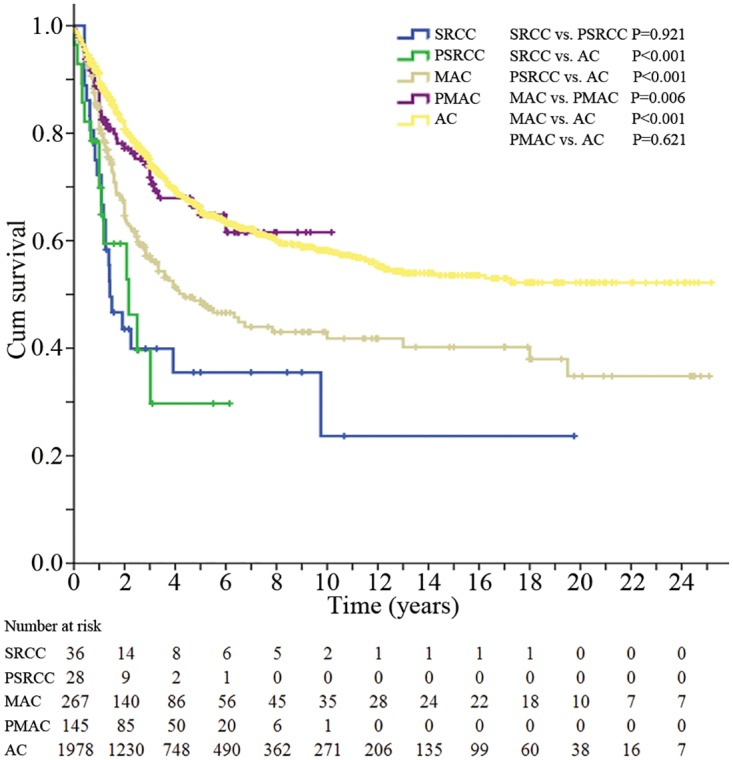
The overall survival of patients in the five groups. SRCC, signet-ring cell carcinoma; PSRCC, partial signet-ring cell carcinoma; MAC, mucinous adenocarcinoma; PMAC, partial mucinous adenocarcinoma; AC, classic adenocarcinoma.

The follow-up period lasted 1–302 months (median 32.7 months). The 5-year CSS and 10-year CSS of the overall population were 62.6% and 55.6%, respectively, as shown in Table 2. The Kaplan-Meier survival curves for these five groups are shown in Fig. 2. The 5-year CSS of SRCC and PSRCC patients were 35.5% and 29.7%, respectively, which were the worst prognoses among the five groups. Next were the MAC patients, with a 5-year CSS of 48.8%. The CSS of SRCC, PSRCC and MAC were all significantly lower than that of AC, and the *P* values were all <0.001. However, the difference in 5-year CSS between PMAC and AC was not significant (*P* = 0.621). A similar situation was observed for 10-year CSS.

**Table 2:** The comparison of 5-year CSS and 10-year CSS in the five groups.

	SRCC (n = 36)	PSRCC (n = 28)	MAC (n = 267)	PMAC (n = 145)	AC (n = 1978)	Total (n = 2454)
5-year CSS	35.50%	29.70%	48.80%	64.80%	65.30%	62.60%
10-year CSS	23.70%	0	41.80%	61.60%	58.20%	55.60%
*P* value*	<0.001	<0.001	<0.001	0.621		<0.001

* results compared with AC. CSS, cancer-specific survival; SRCC, signet-ring cell carcinoma; PSRCC, partial signet-ring cell carcinoma; MAC, mucinous adenocarcinoma; PMAC, partial mucinous adenocarcinoma; AC, classic adenocarcinoma.

Without considering the stage IV patients, the recurrence rate of SRCC patients was 52.0%, which was significantly the highest rate among the five groups (*P* < 0.001). The recurrence rates of PSRCC, MAC, PMAC, and AC patients were 36.4%, 36.7%, 20.9%, and 22.8%, respectively.

### The influence of signet-ring cell on the prognoses of CRC patients

In this section, we focus on the prognostic comparison among SRCC, PSRCC and AC patients. The 5-year CSS of SRCC, PSRCC and AC patients was 35.5%, 29.7% and 65.3%, respectively. Differences between SRCC and AC, PSRCC and AC, were obvious (P<0.001), whereas the difference between SRCC and PSRCC was not significant (P = 0.921). Next, stratified analyses based on age at diagnosis, gender, tumor site and tumor stage were conducted to explore the differences of CSS between SRCC and PSRCC patients in every subgroup. The results are shown in Table 3. The Kaplan-Meier survival curves of each subgroup were shown in S1 Fig. The differences between SRCC and PSRCC were not significant in any of the stratified analyses. Significant differences were found in most of the stratified analyses when comparing SRCC or PSRCC with AC. However, there were several exceptions, including the comparison between young PSRCC patients and young AC patients (*P* = 0.510), female PSRCC and female AC (*P* = 0.058), left-sided colon SRCC and left-sided colon AC (*P* = 0.078), rectal PSRCC and rectal AC (*P* = 0.109), and high-stage PSRCC and high-stage AC (*P* = 0.054). These exceptions may be a result of a limited number of cases in these categories. For example, only 4 cases were available in the young PSRCC group.

**Table 3:** Stratified analysis of 5-year CSS with age at diagnosis, gender, tumor site and tumor stage in SRCC and PSRCC patients.

	5-year CSS			*P* value			
Variance	SRCC (36)	PSRCC (28)	AC (1978)	SRCC vs. PSRCC	SRCC vs. AC	PSRCC vs. AC	Total
**Total**	35.50%	29.70%	65.30%	0.921	<0.001	<0.001	
**Age**							
< = 35yrs	20.50%	37.50%	57.90%	0.291	0.001	0.51	0.004
>35yrs	44.30%	27.20%	65.60%	0.308	0.005	<0.001	<0.001
**Gender**							
Male	36.30%	22.90%	65.60%	0.434	<0.001	<0.001	<0.001
Female	36.90%	42.40%	64.80%	0.698	0.002	0.058	0.002
**Tumor site**							
Right-sided colon	29.60%	20.00%	68.20%	0.583	<0.001	0.002	<0.001
Left-sided colon	37.00%	52.50%	70.00%	0.522	0.078	0.002	0.002
Rectum	35.60%	41.70%	61.90%	0.75	<0.001	0.109	0.001
**Tumor stage**							
Stage I-II	66.70%	N	82.00%	N	0.048	N	0.048
Stage III-IV	28.20%	29.70%	44.90%	0.746	0.009	0.054	0.007
**Surgery**							
R0 resection	50.20%	44.20%	73.70%	0.944	<0.001	<0.001	<0.001
R1 resection	0	0	13.50%	0.125	0.57	0.019	0.054
R2 resection	0	N	4.20%	N	0.794	N	0.794

CSS, cancer-specific survival. N, data cannot be figured out. SRCC, signet-ring cell carcinoma; PSRCC, partial signet-ring cell carcinoma; AC, classic adenocarcinoma.

Multivariate analysis using the Cox proportional hazards model showed that only SRCC, PSRCC, tumor stage at presentation, R1 resection and R2 resection were independent indicators of poor survival (HR, 1.699, *P* = 0.016; HR, 2.182, *P* = 0.005; HR, 2.419, *P* < 0.001; HR, 4.431, *P* < 0.001; HR, 10.085, *P* < 0.001, respectively), while left-sided colon was an independent protective indicator (HR, 0.745, *P* = 0.004). (Table 4)

**Table 4:** Multivariate analysis using Cox’s model for SRCC, PSRCC and AC.

Variance	Hazard ratio	95.0% Confidence interval	*P* value
**Tumor stage**			
Stage I-II	1		
Stage III-IV	2.419	1.993–2.937	<0.001
**Histologic type**			
AC	1		
SRCC	1.699	1.105–2.610	0.016
PSRCC	2.182	1.269–3.751	0.005
**Age**			
< = 35years old	1		
>35years old	0.783	0.576–1.065	0.119
**Gender**			
Male	1		
Female	0.983	0.838–1.154	0.836
**Tumor site**			
Rectum	1		
Right-sided colon	0.854	0.700–1.042	0.12
Left-sided colon	0.745	0.611–0.909	0.004
**Surgery**			
R0 resection	1		
R1 resection	4.431	3.604–5.447	<0.001
R2 resection	10.085	7.333–13.869	<0.001

Table 4 CSS, cancer-specific survival. SRCC, signet-ring cell carcinoma; PSRCC, partial signet-ring cell carcinoma; AC, classic adenocarcinoma.

### The influence of mucin on the prognoses of CRC patients

In this section, we focus on the prognostic comparison of MAC, PMAC and AC. The 5-year CSS of MAC, PMAC and AC patients were 48.8%, 64.8% and 65.3%, respectively. The difference between MAC and AC was obvious (*P* < 0.001), while the difference between PMAC and AC was not significant (*P* = 0.621). PMAC patients had a much better 5-year CSS than did MAC patients (*P* = 0.006). The results of stratified analyses based on age at diagnosis, gender, tumor site and tumor stage are shown in Table 5. The Kaplan-Meier survival curves of each subgroup were shown in S2 Fig. The differences between MAC and AC were all significant in all the stratified analyses, while none of the differences between PMAC and AC was significant. In the comparison between MAC and PMAC, almost no significant difference was found in the stratified analyses, except that male MAC patients, rectal MAC patients and high-stage MAC patients had markedly lower 5-year CSS than the corresponding PMAC patients (*P* = 0.012, *P* = 0.018 and *P* = 0.047, respectively).

**Table 5:** Stratified analysis of 5-year CSS with age at diagnosis, gender, tumor site and tumor stage in MAC and PMAC patients.

	5-year CSS			*P* value			
Variance	MAC (267)	PMAC (145)	AC (1978)	MAC vs. PMAC	MAC vs. AC	PMAC vs. AC	Total
**Total**	48.80%	64.80%	65.30%	0.006	<0.001	0.621	
**Age**							
< = 35yrs	32.40%	N	57.90%	0.077	0.006	0.218	0.007
>35yrs	51.70%	63.90%	65.60%	0.054	<0.001	0.418	<0.001
**Gender**							
Male	53.50%	69.60%	65.60%	0.012	0.002	0.492	0.004
Female	43.30%	56.40%	64.80%	0.31	<0.001	0.076	<0.001
**Tumor site**							
Right-sided colon	46.90%	66.70%	68.20%	0.268	0.005	0.367	0.017
Left-sided colon	54.30%	62.30%	70.00%	0.214	0.004	0.563	0.018
Rectum	45.30%	64.20%	61.90%	0.018	<0.001	0.662	<0.001
**Tumor stage**							
Stage I-II	70.80%	85.00%	82.00%	0.1	0.027	0.68	0.072
Stage III-IV	31.10%	46.70%	46.00%	0.047	<0.001	0.883	0.001
**Surgery**							
R0 resection	57.90%	72.00%	73.70%	0.019	<0.001	0.596	<0.001
R1 resection	0	7.60%	13.50%	0.581	0.023	0.37	0.063
R2 resection	11.10%	N	4.20%	N	0.352	N	0.352

N, data cannot be figured out. CSS, cancer-specific survival. MAC, mucinous adenocarcinoma; PMAC, partial mucinous adenocarcinoma; AC, classic adenocarcinoma.

Multivariate analysis using the Cox proportional hazards model showed that MAC, tumor stage at presentation, R1 resection and R2 resection were independent predictors with decreased survival (HR, 1.532, *P* < 0.001; HR, 2.496, *P* < 0.001; HR, 4.474, *P* < 0.001; HR, 8.186, *P* < 0.001,respectively), but PMAC was not (HR, 1.137, *P* = 0.431). (Table 6).

**Table 6:** Multivariate analysis using Cox’s model for MAC, PMAC and AC.

Variance	Hazard ratio	95.0% Confidence interval	*P* value
**Tumor stage**			
Stage I-II	1		
Stage III-IV	2.496	2.094–2.974	<0.001
**Histologic type**			
AC	1		
MAC	1.532	1.255–1.870	<0.001
PMAC	1.137	0.826–1.566	0.431
**Age**			
< = 35years old	1		
>35years old	0.821	0.627–1.074	0.15
**Gender**			
Male	1		
Female	1.043	0.903	1.205
**Tumor site**			
Rectum	1		
Right-sided colon	0.871	0.728–1.041	0.128
Left-sided colon	0.778	0.650–0.931	0.006
**Surgery**			
R0 resection	1		
R1 resection	4.474	3.707–5.401	<0.001
R2 resection	8.186	6.095–10.995	<0.001

CSS, cancer-specific survival. MAC, mucinous adenocarcinoma; PMAC, partial mucinous adenocarcinoma; AC, classic adenocarcinoma.

## Discussion

Our study identified the different clinicopathological characteristics and CSS of five groups of patients. With respect to overall outcome, SRCC and PSRCC patients had similar CSS and the worst prognoses among the five groups. The next group was MAC, and the best groups were patients with PMAC and AC. There was no significant difference between PMAC and AC. After stratified and multivariate analyses, SRCC, PSRCC and MAC were independent predictors of decreased survival, while PMAC was not.

Only an adenocarcinoma with a substantial amount (≥50%) of signet-ring cells (or mucin) retained within the tumor would be defined as SRCC (or MAC), according to the WHO criterion. However, in clinical practice, colorectal adenocarcinomas with the presence of signet-ring cells (or mucin) in < 50% of the tumor and adenocarcinomas without any signet-ring cells (or mucin) are usually classified as non-signet-ring cell, non-mucinous carcinoma. Therefore, in our study, we divided these tumors into the categories of PSRCC, PMAC and AC to investigate the differences between them.

Our study showed that PSRCC tumors behaved in a pattern similar to that of SRCC tumors. PSRCC and SRCC had similar clinicopathological characteristics and CSS. The stratified and multivariate analyses showed that PSRCC was independently associated with the same poor prognosis that SRCC was. This similarity between SRCC and PSRCC was not surprising, given that SRCC is widely known to be a highly aggressive subtype of CRC. The difference between SRCC and PSRCC is the relative proportion of signet-ring cells in the tumor. Accordingly, the signet-ring cells may affect the overall outcome of the whole tumor.

To date, very few studies on clinicopathological characteristics and overall outcome of PSRCC have been reported. Pande et al retrospectively compared the outcome and pattern of metastases among PSRCC, MAC, PMAC and AC patients [1]. Their results showed no significant difference in the survival of stage IV patients between these groups, which might be due to the limited number of cases in their study. In our study, the 5-year CSS of PSRCC patients in high stages (stage III and IV) was lower than that of high-stage AC patients (29.7% vs. 44.9%), and the difference between them was almost significant (*P* = 0.054). Two other studies have noted that patients with mucinous adenocarcinoma with signet-ring cells had a significantly worse survival rate than patients with mucinous adenocarcinoma without signet-ring cells [11,28]. Similarly, in the CAIRO2 (Capecitabine, Irinotecan and Oxaliplatin in Advanced Colorectal Cancer Trial 2) study, MAC patients without signet-ring cell differentiation had longer median CSS than MAC patients with signet-ring cell differentiation [29]. Finally, a study demonstrated that the molecular features of CRC with a minor (<50%) signet ring cell were similar to those of signet ring cell carcinoma [30]. Therefore, PSRCC and SRCC should be equally important in the overall outcome of CRC patients, and we suggest that the presence of signet-ring cells be noted in pathological reports.

For PMAC, the clinicopathological characteristics and overall outcome were similar to those for AC, but not MAC. These results run counter to the relationship between PSRCC and SRCC. After stratified and multivariate analyses, PMAC was not an independent predictor of the overall outcome. The influence of mucin on the prognoses of CRC patients was different from that of signet-ring cells. However, in the female subgroup analysis, we found that the outcome of PMAC tended to be close to that of MAC, instead of AC. This result may be attributed to the fact that MAC was associated with relatively poor prognoses among female patients when compared with the prognoses of male patients, which was a finding of our study.

Most of the previous researches on this topic have focused primarily on MAC, not PMAC, and the importance of mucin on prognosis has not been well established. One study included stage II and III CRC patients who were treated with adjuvant FOLFOX chemotherapy. It showed that only MAC had an adverse prognostic impact whereas PMAC patients had better disease-free survival (DFS), similar to that for CRC patients without any mucin [31]. Multivariate analysis revealed MAC, not PMAC, to be an independent negative prognostic factor of DFS. Another recent study, by Kim et al, only included patients with stage III colon cancer who were treated with adjuvant FOLFOX chemotherapy [32]. In that study, the clinicopathological characteristics of PMAC were similar to those of MAC. PMAC and MAC were merged into one group for the purpose of calculating the 3-year DFS, which was significantly worse than for AC. The authors concluded that the merged group (PMAC and MAC) was associated with decreased DFS in multivariate analyses. By contrast, a study by Langner et al that included MAC, PMAC and AC patients showed that the presence of extracellular mucin, regardless of its extent in tumor, did not have an effect on patient outcome [33]. This result was assessed by univariate and multivariate analyses. A similar result was obtained in an early study performed by Halvorson et al [28].

The prognostic significance of MAC in CRC patients is still controversial. In our study, after stratified analyses and adjustment for tumor stage, age at diagnosis, gender and tumor site, MAC was found to be an independent predictor of poor prognosis. This finding is consistent with several previous studies, including three retrospective studies in CRC patients treated with first-line chemotherapy, showing that patients with mucinous histology had much worse prognoses than those with the non-mucinous subtype [31,34–37]. However, in some studies [2,7,18,38], after subgroup analysis and multivariate analysis, MAC was not an independent adverse prognostic factor, but instead was related to tumor stage or specific tumor site. For instance, a study based on the data from National Cancer Data Base (NCDB) found that MAC of the rectum, but not MAC of the colon, was associated with poor outcomes [7]. Another study from the Surveillance, Epidemiology, and End Results (SEER) database of the National Cancer Institute suggested that MAC was an independent poor survival indicator in rectal cancer and an independent protective survival indicator in patients with right-sided colon disease, but no significant association of MAC with survival indicators was found in patients who had left-sided colon disease[10].

To our knowledge, our study is the largest to analyze the influence of signet-ring cells and mucin on the prognoses of CRC patients. However, the study had some limitations. The clinical data we collected did not include the signs and symptoms, performance status, carcinoembryonic antigen (CEA), lactate dehydrogenase, approach of surgery, other therapy options (chemotherapy, radiotherapy, targeted therapy or traditional Chinese medicine) and molecular features, such as MSI status and BRAF mutation, which may have some influence on the overall outcome. The second limitation was the selection bias of the patients, as they were all treated in a single institution. Most of the patients were admitted for surgery, but for some of the stage IV patients in the outpatient department, it would have been inadvisable for them to have anti-tumor therapy, or they may have refused to accept treatment. Accordingly, such patients would not have been included in our study. From the existing data, we can see that SRCC, PSRCC and MAC patients had more advanced stage disease at presentation. The incidence of SRCC, PSRCC and MAC in the population who refused the treatment may therefore be higher than that observed in our study.

## Conclusion

Our retrospective study included 2454 colorectal patients treated in our hospital. This study may be the largest report to examine the clinicopathological characteristics and the influence of signet-ring cell and mucinous histology on the prognoses of CRC patients. The age at diagnosis, the tumor sites, T stage, N stage, M stage, and TNM stage varied among SRCC, PSRCC, MAC, PMAC and AC. Compared with AC, SRCC, PSRCC and MAC occurred more frequently in young patients and in high TNM stage. Our study emphasized the crucial role of signet-ring cells on the prognoses of CRC patients, regardless of extent. For patients with a component of mucin, only the MAC patients with ≥50% of mucin had poor prognoses, while those with < 50% mucin had a similar survival to AC patients. In clinical practice, patients with a component of signet-ring cells, regardless of the extent, or ≥50% mucin, should be given more clinical attention.

## Supporting Information

S1 Fig The stratified comparisons of overall survival among SRCC, PSRCC and AC patients. (TIF)

S2 Fig The stratified comparisons of overall survival among MAC, PMAC and AC patients. (TIF)
