# Supplementary figures and images for: A Minor (<50%) Signet-Ring Cell Component Associated with Poor Prognosis in Colorectal Cancer Patients: A 26-Year Retrospective Study in China
Source: PLoS One. 2015 Mar 19;10(3):e0121944. doi: 10.1371/journal.pone.0121944 (PMC4366148; doi:10.1371/journal.pone.0121944)

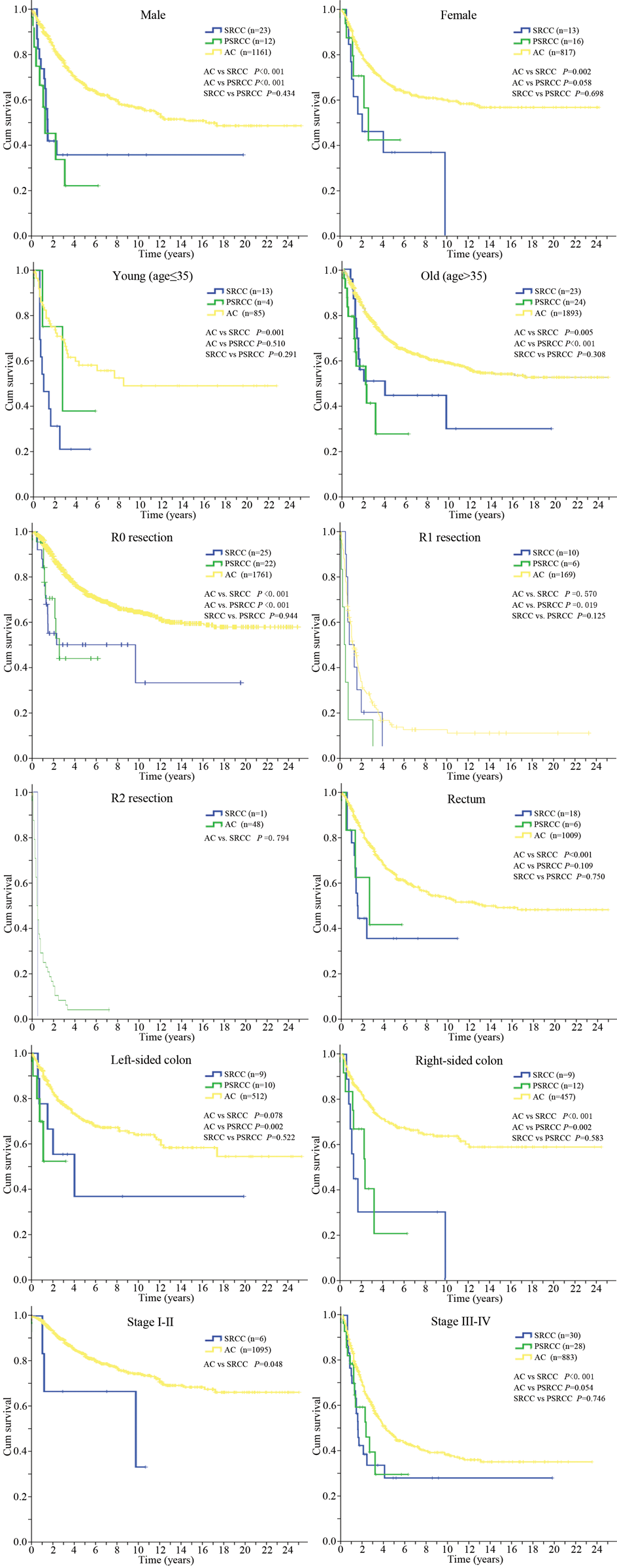

Supplement: S1 Fig — (TIF) [file pone.0121944.s001.tif]

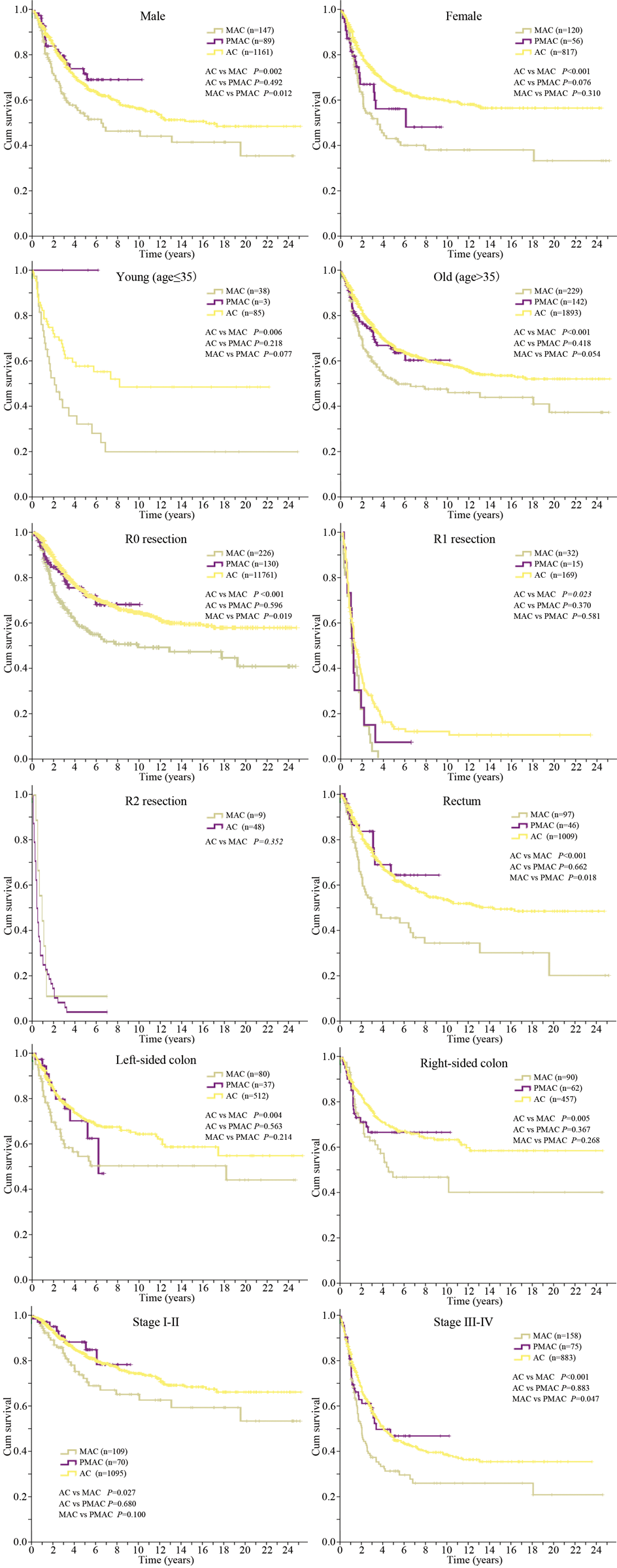

Supplement: S2 Fig — (TIF) [file pone.0121944.s002.tif]
